# Nutritional Support and Clinical Outcome of Severe and Critical Patients With COVID-19 Pneumonia

**DOI:** 10.3389/fnut.2020.581679

**Published:** 2020-11-19

**Authors:** Xiangfeng Yue, Min Li, Yu Wang, Jing Zhang, Xinyi Wang, Linwei Kan, Xiaojian Zhang, Shuzhang Du

**Affiliations:** ^1^Department of Pharmacy, The First Affiliated Hospital of Zhengzhou University, Zhengzhou, China; ^2^Department of Pharmacy, Zhengzhou University, Zhengzhou, China

**Keywords:** nutrition support, protein, severe and critical patients, 2019-nCoV pneumonia, permissive low calorie intake

## Abstract

**Background:** In 2020, a novel coronavirus has spread throughout the world. More than four hundred thousand people have died of SARS-CoV-2 pneumonia, most of which were severe and critical patients. No effective antiviral treatment has been verified thus far. Nutrition support has become one of the important treatments for severe and critical patients.

**Methods:** In this retrospective study, 26 severe patients and 22 critical patients with laboratory confirmed COVID-19 were enrolled. We recorded the diet and nutritional treatments in severe and critical patients. Baseline characteristics and clinical outcomes of severe and critical patients were also collected.

**Results:** Average calorie intake of severe patients (19.3 kcal/kg/d) was higher than critical patients (15.3 kcal/kg/d) (*P* = 0.04). Protein intake was similar in the two groups (0.65 and 0.62 g/kg per day, respectively; *P* = 0.29). There was no significant difference in the median duration of viral shedding between the severe and critical patients (*P* = 0.354).

**Conclusions:** A permissive underfeeding strategy that restricts non-protein calories but preserves protein intake is feasible for critical patients with SARS-CoV-2 pneumonia. Viral shedding duration of critical patients was the same as severe patients who received standard feeding. Nevertheless, evidence of the conclusion is not sufficient because of small sample size. To show the real clinical benefit of permissive low-calorie and adequate protein intake in critical SARS-CoV-2 pneumonia patients, a large and pragmatic randomized controlled trial is needed.

## Introduction

In December, 2019, a series of cases of pneumonia associated with a novel coronavirus, SARS-CoV-2, emerged in China. The novel coronavirus pneumonia was declared as a global pandemic by World Health Organization (WHO) director general, Tak Desai, in March 11, 2020. So far, over 9 million people have been infected, and four hundred thousand people have died of COVID-19 (1). According to diagnosis and treatment of novel coronavirus pneumonia (trial version seventh), SARS-CoV-2 pneumonia can be classified as light, general, severe, and critical types (2). Most patients have mild symptoms and good prognosis while some severe and critical cases may appear as acute respiratory distress syndrome or septic shock, even death (3). A large number of medical resources were invested in the treatment of severe and critical cases. Compared with that of severe patients, the progress of critical patients was rapid and difficult to control. At present, no effective antiviral drug is available against this pathogen. Life support is the main treatment for severe and critical patients with SARS-CoV-2 pneumonia (4).

Respiratory support and clinical nutritional support are important parts of supportive therapy for severe and critical patients. It is well-known that a nutrition risk score (NRS2002) ≥3 would result in poor prognosis (5). Up to now, studies on SARS-CoV-2 pneumonia have mainly focused on the clinical characteristics and treatment process of patients (6, 7). Previous studies have reported that high risk factors for death in patients with SARS-CoV-2 pneumonia are older age, high SOFA score, and d-dimer >1 μg/L (8). So far, no study has discussed the relationship between nutritional support and clinical outcomes in severe and critical patients infected COVID-19.

In this paper, we described baseline characteristics, nutritional support, and clinical outcomes of severe and critical patients. We hope our study results can provide some valuable suggestions on nutritional support of severe and critical patients with SARS-CoV-2 pneumonia.

## Methods and Materials

### Study Design and Participants

Forty-eight patients were recruited from January 29 to March 6, 2020 at a designated hospital for SARS-CoV-2 pneumonia. All patients were diagnosed as having SARS-CoV-2 pneumonia according to WHO interim guidance. Among the 48 patients included, 26 were categorized as severe type, and 22 were critical type. We calculated the nutritional intake of severe and critical patients and analyzed the relationship between the nutrition supply and clinical outcomes. The assigned time was 7 days from admission into the intensive care unit (ICU) and discharge. If the ICU stay time is <7 days, assigned time is from the first day in ICU until discharge. The primary outcome was the time of COVID-19 viral clearance. This study was approved by the research project ethics committee in our hospital (2020-KY-115).

### Data Collection

The data of treatments and outcomes were extracted from electronic medical records using a standard data collection statistical analysis form (modified case record form for severe acute respiratory infection clinical characterization shared by the International Severe Acute Respiratory and Emerging Infection Consortium). We directly contacted doctors or nurses to obtain the missing data. All data were checked by two physicians (YXF and LM), and a third researcher (WY) adjudicated any difference in interpretation between the two primary reviewers.

### Definitions

Critical patients met one of the following conditions (2): respiratory failure and need for mechanical ventilation or shock combined with other organ failure and need for ICU monitoring and treatment.

Severe patients meet one of the following conditions (2): respiratory distress or respiratory frequency ≥30 times/min, The oxygen saturation is ≤93%, PaO_2_/FiO_2_ × [atmospheric pressure (mmHg)/760] should be corrected according to the following formula in the area with PaO_2_/FiO_2_ ≤300 mmHg (1 mmhg = 0.133 kpa) (altitude over 1,000 m). In addition, if pulmonary imaging shows that the lesions have progressed more than 50% within 24–48 h, the patient can also be classified as severe.

### Statistical Analysis

Continuous variables were presented as mean (SD) if they are normally distributed or median (IQR) if they are not. Categorical variables were expressed as number (%). Mann-Whitney *U*-test, independent sample *t*-test, χ^2^-test, or Fisher's exact test was used to compare differences between severe and critical patients where appropriate. Boxplots were drawn to describe calorie intake and protein intake. Statistical analyses were done using SPSS software (version 21.0).

### Role of the Funding Source

The funder of the study had no role in study design, data collection, data analysis, data interpretation, or writing of the report. The corresponding authors had full access to all the data in the study and had final responsibility for the decision to submit for publication.

## Results

### Baseline Characteristics

All of the 48 patients involved in this study were identified as laboratory-confirmed SARS-CoV-2 infection. They were admitted to ICU because of uncorrectable hypoxemia. There was no difference in age (*P* = 0.197), sex (*P* = 0.514), weight (*P* = 0.25), and body mass index (BMI) (*P* = 0.379) between severe and critical patients at the time of admission. All patients had nutrition risk (NRS2002 score ≥3). The NRS2002 scores of critical patients mainly were 5–7 (21[95.5%]), while the severe patients were 3–4 (20 [76.9%]). The NRS2002 scores of critical patients were higher than those of severe patients (*P* < 0.0001) (Table 1).

**Table 1:** Baseline characteristics of patients included^*^.

**Variable**	**Total (48)**	**Severe (*n =* 26)**	**Critical (*n =* 22)**	***P-*value**
Age: years	61.9 ± 15.8	59 ± 15.4	65 ± 15.8	0.16
Sex: number (%)				0.312
Male	29 (60.4)	15 (57.7)	13 (59.1)	
Female	19 (39.6)	11 (42.7)	9 (40.9)	
Weight: kg	68.1 ± 8.1	68.1 ± 9.1	68.2 ± 7.1	0.501
Body mass index^†^	24.6 ± 2.3	24.7 ± 2.6	24.5 ± 2.0	0.682
NRS2002 score				<0.0001
5–7	27 (56.3)	6 (23.1)	21 (95.5)	
3–4	21 (43.8)	20 (76.9)	1 (4.5)	
Admission to ICU from illness onset: days (IQR)^‡^	16.5 (10.0–20.8)	18.5 (10.8–23.0)	14.5 (10.0–18.3)	0.259
Invasive mechanical ventilation: number (%)	12 (24)	0	12(54.5)	<0.0001
Hemodialysis: number (%)	1 (4.8)	0	1(4.5)	0.458
**Complications: number (%)**				
Hypertension	16 (33.3)	10 (38.5)	6 (27.3)	0.413
Diabetes	11 (22.9)	4 (15.4)	7 (31.8)	0.177
Coronary heart disease	8 (16.7)	4 (15.4)	4 (18.2)	1
COPD	3 (6.2)	1 (3.8)	2 (9.1)	0.587
Cancer and immune diseases	4 (8.3)	3 (11.5)	1 (4.5)	0.614
Chronic renal failure	2 (4.2)	1 (3.8)	1 (4.5)	1

* *Plus–minus values are means ± SD*.

† *The body–mass index is the weight in kilograms divided by the square of the height in meters*.

‡ *IQR means interquartile range*.

The median time from illness onset to ICU admission was 16.5 days (10.0–20.8), and there was no difference between critical and severe patients (*P* = 0.259) (Table 1). Comorbidities were not present in only 10 patients. Hypertension was the most common chronic disease (16[33.3%]), followed by diabetes, coronary heart disease, chronic obstructive pulmonary disease (COPD), cancer and immune system diseases, and chronic renal failure. Twelve patients required invasive mechanical ventilation, and one patient needed hemodialysis before admission to the ICU (Table 1).

### Life and Nutrition Support

Twenty-three patients (47.9%) received invasive mechanical ventilation, 15 patients (31.3%) received extracorporeal membrane oxygenation (ECMO) support, and 11 patients (22.9%) received hemodialysis until discharged from ICU. The percentage of critical patients who needed life support was markedly higher than critical patients (Table 2).

**Table 2:** The included patients' nutrition and life support for a duration of 7 days^*^.

**Variable**	**Total (*n =* 48)**	**Severe (*n =* 26)**	**Critical (*n =* 22)**	***P*-value**
Daily calorie intake: kcal/d	1202.2 ± 365.1	1306.6 ± 329.6	1082.1 ± 374.8	0.04
Average calorie intake: kcal/kg/d	17.4 ± 5.6	19.3 ± 5.4	15.3 ± 5.1	0.018
Daily protein intake: g/d	43.2 ± 17.6	39.0 ± 12.7	47.7 ± 21.1	0.106
Average protein intake: g/kg/d	0.64 ± 0.25	0.65 ± 0.21	0.62 ± 0.29	0.076
Invasive mechanical ventilation: number (%)	23 (47.9)	6 (23.1)	17 (77.3)	<0.0001
ECMO: number (%)^†^	15 (31.3)	4 (15.4)	11 (50)	0.01
Hemodialysis: number(%)	11 (22.9)	1 (3.8)	10 (45.5)	0.001
**Gastrointestinal complications: number (%)**				
Diarrhea: number (%)^‡^	13 (27.1)	6 (23.1)	7 (31.8)	0.497
Feeding intolerance: number (%)^§^	5 (10.4)	0	5 (22.7)	0.015

* *Plus–minus values are mean ± SD*.

† *ECMO is extracorporeal membrane oxygenation*.

‡ *Diarrhea was defined as three or more loose or liquid stools per day for 2 consecutive days*.

§ *Feeding intolerance was defined as vomiting, abdominal distention, or a gastric residual volume of more than 200 ml*.

**Figure 1:**
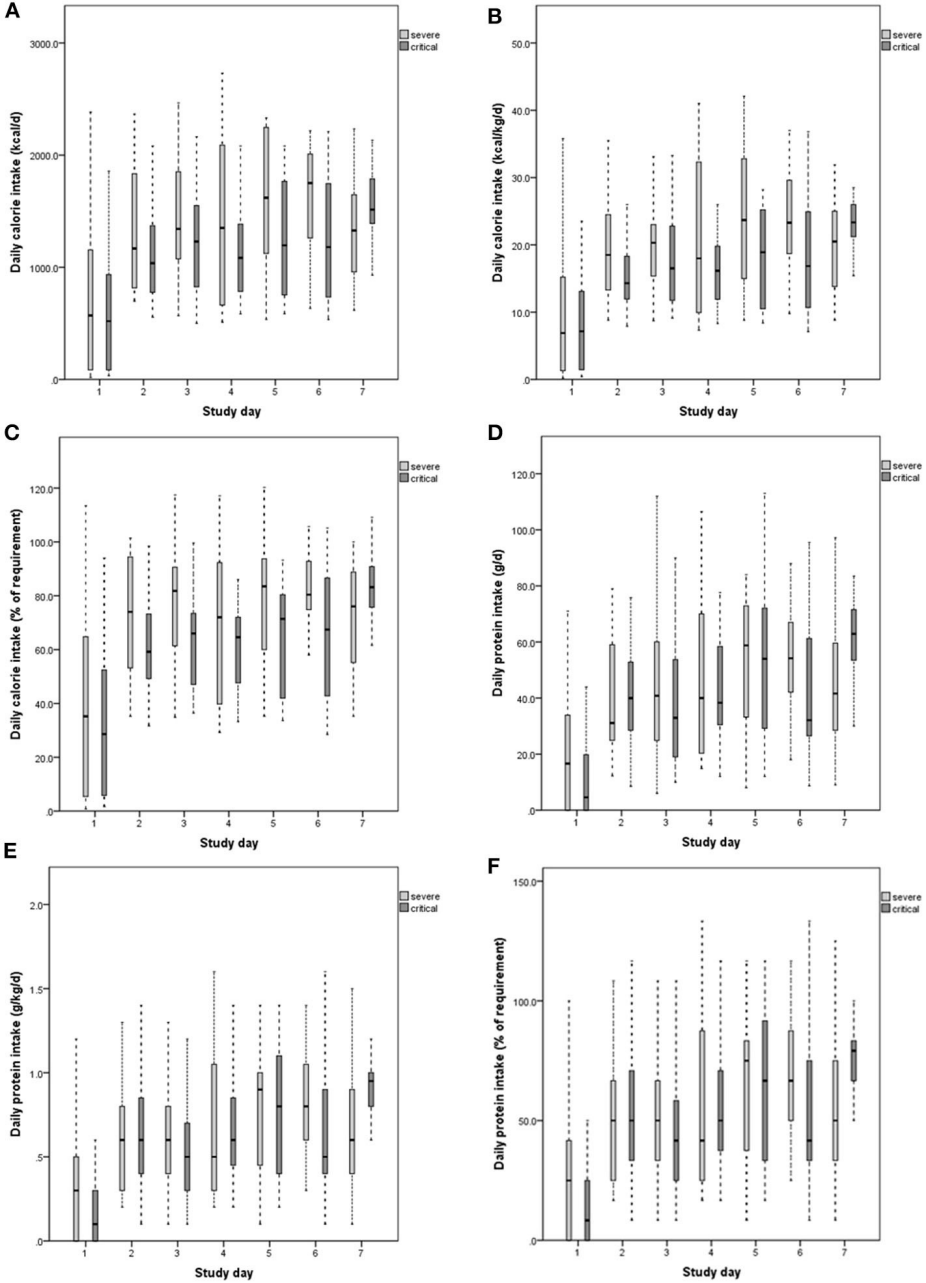
Daily calorie intake **(A–C)**, protein intake **(D–F)** of severe and critical patients 7 days after admission to ICU (from day 0 to day 7). The calorie intake requirement is 25 kcal/kg/d. The protein intake requirement is 1.2 g/kg/d. The top panels show the amount of calories administered daily over 7 days after admission to ICU, expressed in kcal/d **(A)** and kcal/kg/d **(B)**. **(C)** Shows percentages of calorie intake compared with the required level. Protein intake was expressed in g/d **(D)** and d/kg/d **(E)**. **(F)** Shows percentages of protein intake compared with the required level.

The average calorie intake of severe and critical patients was 19.3 kcal/kg/d (5.4) and 15.3 kcal/kg/d (5.2), respectively. There was a significant difference between the two types of patients (*P* = 0.018). The average protein intake of severe and critical patients was 0.65 g/kg/d (0.21) and 0.62 g/kg/d (0.29), respectively. A significant difference was not observed between the two types of patients (*P* = 0.076). The calorie intake per day of severe patients was 1306.6 kcal (329.6), which was higher than that of critical patients (1082.1 kcal [374.8], *P* = 0.04). The protein intake per day of severe patients was 39.0 g (12.7), which was lower than that of critical patients (47.7 g [21.1]). A significant difference was also not observed (*P* = 0.106) (Table 2). The 7-day calorie and protein intake in severe and critical patients are shown in Figure 1.

Compared with that of the severe patients, oral intake for critical patients was more affected: 11 severe patients (42.3%) and 15 critical patients (68.2%) received enteral nutrition support. The incidence of gastric retention was higher in critically patients (*P* = 0.015). There was no difference in the incidence of diarrhea between the two types of patients (Table 2).

### Clinical Outcomes and Clinical Indicators

The nucleic acid test was negative in 45 patients (93.8%) while 3 critical patients (6.2%) died before the viral clearance until ICU discharge. The nucleic acid test was negative in 20 (90.9%) critical patients and 25 (96.2%) severe patients (*P* = 0.587). The median duration of viral shedding in severe patients was 21.0 days (IQR 16.0–25.0) from illness onset and 24.0 days (IQR 10.0–20.8) in critical patients. The median time of viral clearance of the critical and severe patients had no significant difference (*P* = 0.354) (Table 3).

**Table 3:** Clinical outcome and clinical indicators of severe and critical patients^*^.

**Variable**	**Total (*n =* 48)**	**Severe (*n =* 26)**	**Critical (*n =* 22)**	***P*-value**
Ratio of viral shedding–: number (%)	45 (93.8)	25 (96.2)	20 (90.9)	0.587
Median duration of viral shedding: –days (IQR)^†^	21.0 (16.0–27.0)	21.0 (16.0–25.0)	24.0 (10.0–20.8)	0.354
**Clinical indicators**				
Serum phosphorus: –mmol/L	0.90 ± 0.19	0.88 ± 0.20	0.92 ± 0.18	0.51
Uric acid: –μmol/L	188.4 ± 90.6	215.5 ± 103.0	158.4 ± 64.6	0.047
Cholinesterase: –KU/L	4.72 ± 1.91	5.41 ± 1.82	3.92 ± 1.75	0.016
Lactic dehydrogenase: –U/L	438.8 ± 254.4	326.8 ± 108.9	563.2 ± 310.6	0.001
Lymphocyte absolute value: −10^9^/L	0.57 ± 0.25	0.64 ± 0.22	0.49 ± 0.26	0.06

* *Plus–minus values are means ± SD*.

† *IQR means interquartile range*.

Certain clinical indicators reflect the nutrition status of patients, such as blood phosphorus, uric acid, cholinesterase (Table 3). There was no significant difference in blood phosphorus (*P* = 0.51) between the severe (0.88 [0.20]) and critical patients (0.92 [0.18]). The uric acid (215.5 [103.0]) in severe patients was higher (*P* = 0.047) than that of critical patients (158.4 [64.6]). Cholinesterase (5.41 [1.82]) in severe patients was significantly higher (*P* = 0.016) than that of critical patients (3.92 [1.75]). Laboratory markers were recorded from entering the ICU. Compared with that of the severe group (326.8 [108.9]), lactate dehydrogenase (LDH) in the critical group (563.2 [310.6]) was significantly higher (*P* = 0.001). There was no significant difference in the absolute value of lymphocytes between the severe patients and the critical patients (*P* = 0.06) (Table 3).

## Discussion

The risk factors of poor prognosis and high mortality in patients with SARS-CoV-2 pneumonia infection are old age and multiple basic diseases (8). Therefore, we used the NRS2002 (inpatient) screening tool to measure nutrition risk. Both severe and critical patients with SARS-CoV-2 pneumonia had nutrition risk (NRS2002 score ≥3). Moreover, recent studies have shown that patients with high FM have poor prognosis (9). It should be noted that BMI leaves a portion of the obese population unrecognized when using the NRS2002 (inpatient) screening tool. Acute respiratory distress syndrome (ARDS) was one of the common serious complications of COVID-19 infected critical patients, which required invasive mechanical ventilation. However, invasive mechanical ventilation will consume a lot of calories and further increase the incidence of malnutrition. Special life supports, such as continuous renal replacement therapy (CRRT) and extracorporeal membrane oxygenation (ECMO), may also result in nutrition loss (10, 11). SARS-CoV-2 pneumonia patients often suffered from fever. The increase in body temperature could lead to increased calorie consumption. The albumin level of patients with severe infection was significantly lower than patients without severe infection. Hypoalbuminemia also could lead to edema of the intestinal mucosa, resulting in decreased intake capacity of nutrients. In the early stage of stress, a large number of visceral proteins and skeletal muscles were decomposed to compensate for the lack of calories in the body. The loss of skeletal muscle volume and function would increase the risk of death (12). Malnutrition in ICU patients is correlated with the incidence of infection complications and mortality rate (13).

Therefore, nutritional support has become one of the important treatments for ICU patients. Nutritional support could reduce the loss of endogenous protein caused by stress (14). The European Society for Clinical Nutrition and Metabolism (ESPEN) guidelines for nutrition diagnosis and treatment of critically ill patients suggest that early nutritional intervention should be initiated within 24 h after admission to the ICU for patients with nutrition risk (15). The calorie supply target (25–30 kcal/kg/d) and protein supply target (1.2–2.0 g/kg/d) should be achieved within 3–7 days if hemodynamics are stable (15).

However, it was difficult to achieve the target supply for severe and critical patients with COVID-19. The reasons were as follows. Because of their high-stress state, the critical SARS-CoV-2 pneumonia patients were more likely to suffer from intolerance of enteral nutrition (EN), with conditions such as diarrhea, abdominal distention, constipation, and gastric retention (16). Although reducing the speed of infusion EN can alleviate gastrointestinal intolerance symptoms (20 ~30 mL/h), but the daily supply of EN was decreased. Second, in the early stages of the disease outbreak, conditions in the isolation ward did not allow the implementation of jejunal tube placement and jejunal stoma. Therefore, it was impossible for the patients with gastric retention to implement jejunal feeding. Supplementary parenteral nutrition (SPN) shall be administrated when the nutrition supply cannot be met by EN (<60% of caloric requirements) for more than seven days (17). However, the brain natriuretic peptides (BNP) of critical SARS-CoV-2 pneumonia patients were generally increased, which led to a higher incidence of heart failure. The total amount of daily liquid intake was limited to reduced cardiac load. Therapeutic drugs took up too much liquid, which restricted the amount of parenteral nutrition. Adoption of ECMO limited the use of fat emulsion, making it more difficult to achieve the sufficient calorie supply through SPN. In addition, due to their stress, infection, hypoxia, and use of glucocorticoids, most severe patients with SARS-CoV-2 pneumonia had different degrees of hyperglycemia. Hyperglycemia could increase the incidence of infection-related complications and finally increased mortality (18). Therefore, enteral nutrition preparations for diabetes of low calorie and protein nutrient density (0.9 kcal ml-1 and 0.038 g kcal-1, respectively) were first used by clinicians, which could not meet the feeding target (25 kcal/kg/d) for patients with SARS-CoV-2 pneumonia.

As nutritional pharmacists, we attempted many methods to solve the aforementioned difficulties during nutritional support. First, a tumor-specific enteral nutritional preparation was applied instead of diabetes preparation, which provided far more calories (1.3 kcal ml^−1^) and protein (0.045 g kcal^−1^). It also had a light impact on blood glucose due to its high dietary fiber and fat content, and low carbohydrate content. Secondly, SPN was started immediately if EN supply was <60% of the caloric target. Meanwhile, the amino acid proportion (≥20% total calories) was higher than normal PN formulation, especially for patients who had received ECMO and CRRT. This was the reason why protein intake was higher in critical patients than severe patients (although no significant difference was observed). Third, we reformed the treatment liquid. Amino acid was infused alone, electrolyte and vitamin were added into 5%glucose. If electrolytes are added to amino acid, the patient's infusion volume will be reduced. To our experience, liquid reforming was very important to critical patients with SARS-CoV-2 pneumonia; it could reduce unnecessary liquid intake and increase liquid for nutritional supply. As a result, the calorie intake of severe and critical patients with SARS-CoV-2 pneumonia reached more than 60% of the recommended amounts for all 7 days except for the first day. The average calorie intake of severe and critical patients was 77.2 and 61.2% of the feeding targets, respectively.

As shown in Table 3, no difference existed in the viral shedding duration and the absolute value of lymph between critical and severe patients. This result was similar to previously reported (8). This may be attributed to a similar protein intake. In 2015, Arabi et al. examined the effect of “permissive low calorie” (40–60% of target calories) and “standard calorie” (70–100% of target calories) on the prognosis of ICU patients with the same amount of protein. The results of this multicenter prospective randomized clinical trial (RCT) studies showed that there was no difference in short- or longterm mortality, sequential organ failure score (SOFA), length of stay, mechanical ventilation time between the two groups (19). Meanwhile, a number of RCT studies showed that the mortality was negatively correlated with protein supply (20). At present, there is no effective treatment for SARS-CoV-2 pneumonia patients. Elimination of the virus mainly depended on the autoimmune system of patients. A recent study reported SARS-CoV-2 pneumonia patients with high lymphocyte levels, excluding B cells, and with low Interleukin-6 (IL-6) or Interleukin-10 (IL-10) levels, had a better survival rate (21). Early nutritional intervention could enhance the therapeutic effect of severe pneumonia patients with ARDS and reduce systemic inflammatory response (22, 23). Its mechanism may be directly related to optimizing nutrition status and improving cellular immune function (24). Protein is the material basis of immune function. When protein intake is insufficient, the structure and function of immune organs could be impaired (25). Permissive low-calorie feeding could reduce the occurrence of metabolic complications. At the same time, sufficient protein intake could promote protein synthesis to make up for the loss of immunoprotein and accelerate the elimination of the virus by the immune system.

## Conclusion

To the best of our knowledge, this is the first retrospective study to report clinical nutrition and life support among severe and critical patients with SARS-CoV-2 pneumonia. This study involved 26 severe patients and 22 critical patients. This paper discusses the status of nutrition support in severe and critical patients with SARS-CoV-2 pneumonia. The differences in calorie intake, protein intake, negative viral infection status, and clinical indexes between severe and critical patients were compared. On the premise of ensuring the metabolic stability of critical patients, adequate nutritional support should be provided to critical patients. Sufficient protein is beneficial for maintaining the amount of lymphocytes, thus promoting virus clearance. Therefore, if adequate calorie intake cannot be achieved, permissive low calorie intake with adequate protein intake is appropriate for critical patients with SARS-CoV-2 pneumonia.

This study has some limitations. The sample size was small, and patients came from one hospital. That there is a real clinical benefit of permissive low-calorie intake and sufficient protein supply in severe SARS-CoV-2 pneumonia patients needs to be further verified by large and pragmatic randomized controlled trials.

## Data Availability Statement

The raw data supporting the conclusions of this article will be made available by the authors, without undue reservation.

## Ethics Statement

The studies involving human participants were reviewed and approved by the research project ethics committee of The First Affiliated Hospital of Zhengzhou University (2020-KY-115). The ethics committee waived the requirement of written informed consent for participation.

## Author Contributions

XY, ML, and SD equally contributed to the conception and design of the research. YW and JZ contributed to the design of the research. LK and XW contributed to the acquisition and analysis of the data. XW contributed to the analysis of the data. XZ contributed to the acquisition, analysis, and interpretation of the data. All authors drafted the manuscript and approved the final manuscript.

## Conflict of Interest

The authors declare that the research was conducted in the absence of any commercial or financial relationships that could be construed as a potential conflict of interest.
